# Varnish for Shoes

**Published:** 1866-12

**Authors:** 


VARNISH FOR SHOES.

It is a bad plan to grease the upper leather of shoes for the purpose of keeping them soft; it rots the leather and admits dampness more readily. It is better to make a varnish thus:

Put half a pound of gum shellac, broken up in small pieces, in a quart bottle or jug, cover it with alcohol, cork it tight and put it on a shelf in a warm place; shake it well several times a day, then add a piece of gum camphor as large as a hen's egg; shake it well, and in a few hours shake it again and add one ounce of lamp black; if the alcohol is good, it will all be dissolved in three days; then shake and use. If it gets too thick, add alcohol—pour out two or three teaspoonfuls in a saucer and apply it with a small paint brush. If the materials were all good, it will dry in about five minutes, and will be removed only by wearing it off, giving a gloss almost equal to patent leather.

The advantage of this preperation above others is, it does not strike into the leather and make it hard, but remains on the surface and yet excludes the water almost perfectly.

This same preperation is admirable for harness, and does not soil when touched, as lamp black preperations do.

